# Comparing Efficacy and Safety of Different Doses of Tirzepatide for the Treatment of Type 2 Diabetes Mellitus: A Meta-Analysis of Randomized Controlled Trials

**DOI:** 10.7759/cureus.44314

**Published:** 2023-08-29

**Authors:** Zarghuna Khan, Muhammad O Naeem, Saad Khalid Khan, Faisal Khan, Muhammad Abdullah, Ilqa Attique, Sana Dur Muhammad, Adil Amin

**Affiliations:** 1 Internal Medicine, Rehman Medical Institute, Peshawar, PAK; 2 Pathology, Rehman Medical Institute, Peshawar, PAK; 3 Medicine, Army Medical College, Rawalpindi, PAK; 4 Medicine, Dow University of Health Sciences, Karachi, PAK; 5 Medicine, Quaid-E-Azam Medical College, Bahawalpur, PAK; 6 Internal Medicine, Foundation University Medical College, Islamabad, PAK; 7 Internal Medicine, Jinnah Sindh Medical University, Karachi, PAK; 8 Cardiology, Pakistan Navy Ship (PNS) Shifa, Karachi, PAK

**Keywords:** meta-analysis, efficacy, safety, type 2 diabetes, tirzepatide

## Abstract

Our study assessed the efficacy and safety of the three primary tirzepatide (TZP) doses, 5 mg, 10 mg, and 15 mg using network meta-analysis to assess their relative impact on type 2 diabetes mellitus (T2DM) treatment. This study adhered to the Preferred Reporting Items for Systematic Reviews and Meta-Analyses (PRISMA) 2020 guidelines. Two authors independently screened online databases, including PubMed, Cochrane Library, and Embase. We employed the keywords "Type 2 diabetes OR T2DM or diabetes" AND "Tirzepatide OR LY3298176 OR twincretin OR dual glucose-dependent insulinotropic polypeptide and glucagon-like peptide-1 receptor agonist" AND "randomized controlled trial". The outcomes evaluated in this study comprised changes in hemoglobin (Hb)A1c levels from baseline (%), changes in weight from baseline (Kg), changes in fasting serum glucose from baseline (mg/dL), and occurrences of serious adverse events (SAE), adverse events (AE) and major adverse cardiovascular events (MACE). A total of eight studies met the inclusion criteria and were included in this meta-analysis. Our findings suggest that among the evaluated doses, TZP at 15 mg demonstrated superior effectiveness in reducing HbA1c, weight, and fasting serum glucose compared to doses of 10 mg and 5 mg. Notably, the reduction in HbA1c and weight showed a dose-dependent trend, with the 15 mg dose achieving the most substantial benefits. The safety analysis indicated that while serious adverse events and major adverse cardiovascular events (MACE) did not significantly differ among the three doses, the risk of overall adverse events was notably higher in the 10 mg and 15 mg TZP groups compared to the 5 mg group.

## Introduction and background

Type 2 diabetes mellitus (T2DM) is characterized by a range of physiological factors, including insulin resistance, impaired insulin secretion, excess body fat, reduced incretin effect, heightened glucagon release, and abnormal lipid levels [1]. Unfortunately, the global prevalence of diabetes among individuals aged 20-79 was 10.5% in 2021, equating to roughly 536.6 million. This figure is projected to rise to 12.2% by 2045, reaching approximately 783.2 million cases [2]. A central element of T2DM is insulin resistance, arising from a combination of factors, with obesity being a significant contributor [3]. In individuals with T2DM, higher adiposity is linked to higher glycemia and increased glycosylated hemoglobin (Hb)A1c levels [4].

Obesity stands as the primary risk factor for type 2 diabetes, playing a key role in insulin resistance, as well as contributing to conditions like hypertension, dyslipidemia, and non-alcoholic fatty liver disease [5-6]. Given their interconnected nature, shedding excess body weight can positively impact blood sugar control, insulin sensitivity, and associated health issues [7]. More substantial weight loss offers the potential for reversing the metabolic irregularities of type 2 diabetes, potentially leading to improved glycemic levels and even diabetes remission [8]. While both insulin and glucagon-like peptide-1 receptor agonists (GLP-1 RAs) have proven effective in glycemic control for T2DM, insulin use carries the risk of hypoglycemia [9]. Moreover, the weight loss efficacy of insulin and GLP-1 RAs remains suboptimal [10], which limits their overall clinical effectiveness. Consequently, there is a pressing need for novel medications that can effectively lower blood sugar levels and promote weight loss in T2DM treatment.

A recent meta-analysis suggests that tirzepatide (TZP) holds promise as a novel hypoglycemic drug for T2DM management [11]. TZP is a dual glucagon-like peptide-1 receptor agonist (GLP-1 RA) and glucose-dependent insulinotropic polypeptide (GIP) that consists of 39 amino acids. It combines a bioactive GIP sequence at the N-terminal and an exenatide-like sequence at the C-terminal while being conjugated with a fatty acid chain resembling semaglutide's side chain. This structural design extends the drug's half-life by enhancing its binding to albumin [12].

A prior meta-analysis revealed that TZP's hypoglycemic effect surpassed that of placebo, insulin, and other GLP-1 RAs [11]. However, this analysis didn't compare the different doses of TZP for T2DM management. Thus, our study directly contrasts the effectiveness and safety of the three primary TZP doses, 5 mg, 10 mg, and 15 mg, using network meta-analysis to assess their relative impact on T2DM treatment. The aim of this meta-analysis is to compare the efficacy and safety of different doses of TZP for the treatment of T2DM.

## Review

Methodology

This study adhered to the Preferred Reporting Items for Systematic Reviews and Meta-Analyses (PRISMA) 2020 guidelines.

Search Strategy and Study Selection

Two authors independently screened online databases, including PubMed, Cochrane Library, and Embase, from their inception dates to August 10, 2023. We employed the keywords "Type 2 diabetes OR T2DM or diabetes" AND "Tirzepatide OR LY3298176 OR twincretin OR dual glucose-dependent insulinotropic polypeptide and glucagon-like peptide-1 receptor agonist" AND "randomized controlled trial". Our search was limited to articles published exclusively in the English language. Furthermore, we manually reviewed the reference lists of all included articles to identify additional studies.

Two authors independently assessed all articles obtained through online searching. After removing duplicates, initial screening involved evaluating titles and abstracts, followed by a detailed review of full texts using predetermined inclusion and exclusion criteria. Any discrepancies during the search and selection process were resolved through consensus.

Inclusion and Exclusion Criteria

We included randomized controlled trials (RCTs) that compared the efficacy and safety of two or more doses of TZP in adult patients with T2DM. We excluded observational studies, case reports, animal studies, reviews, letters, and editorials.

Data Extraction and Outcomes

The following data were extracted: lead author, publication year, study groups, sample size, duration of follow-up, and baseline characteristics of participants, including age, gender, and comorbidities. The outcomes evaluated in this study comprised changes in HbA1c levels from baseline (%), changes in weight from baseline (kg), changes in fasting serum glucose from baseline (mg/dL), and occurrences of serious adverse events (SAE), adverse events (AE) and major adverse cardiovascular events (MACE). One author conducted data extraction. A second author with the included studies subsequently cross-checked. Any disagreements between the two authors were resolved through consensus.

Quality Assessment

Two authors assessed the quality of the studies. The Cochrane Collaboration's risk-of-bias instrument for Randomized Controlled Trials (RCTs) was employed. The seven criteria employed to assess bias in each trial encompassed the creation of randomization sequence, concealment of allocation, masking of participants and staff, masking of outcome evaluations, insufficient outcome data, discriminating reporting, and other predispositions, such as variations in baseline characteristics among distinct groups.

Data Analysis

Meta-analysis was done using STATA 16.0 (StataCorp LLC, College Station, TX) software. The pooled effect sizes were analyzed using a random-effects model. For continuous outcomes, we performed network meta-analysis (NMA) and computed pooled mean difference with 95% confidence interval (CI) among different doses of TZP and for categorical outcomes, we reported odds ratio (OR) with 95% CI. NMA was employed to combine both the direct and indirect impacts. The approach of utilizing NMA extends beyond the typical pairwise meta-analysis, allowing for a concurrent assessment of numerous interventions. This forms an interconnected network while upholding each trial's inherent randomization We calculated the comparative hierarchy of distinct doses of TZP for each result by utilizing the distribution of ranking probabilities alongside the surface under the cumulative ranking curves (SUCRA).

Results

**Figure 1:**
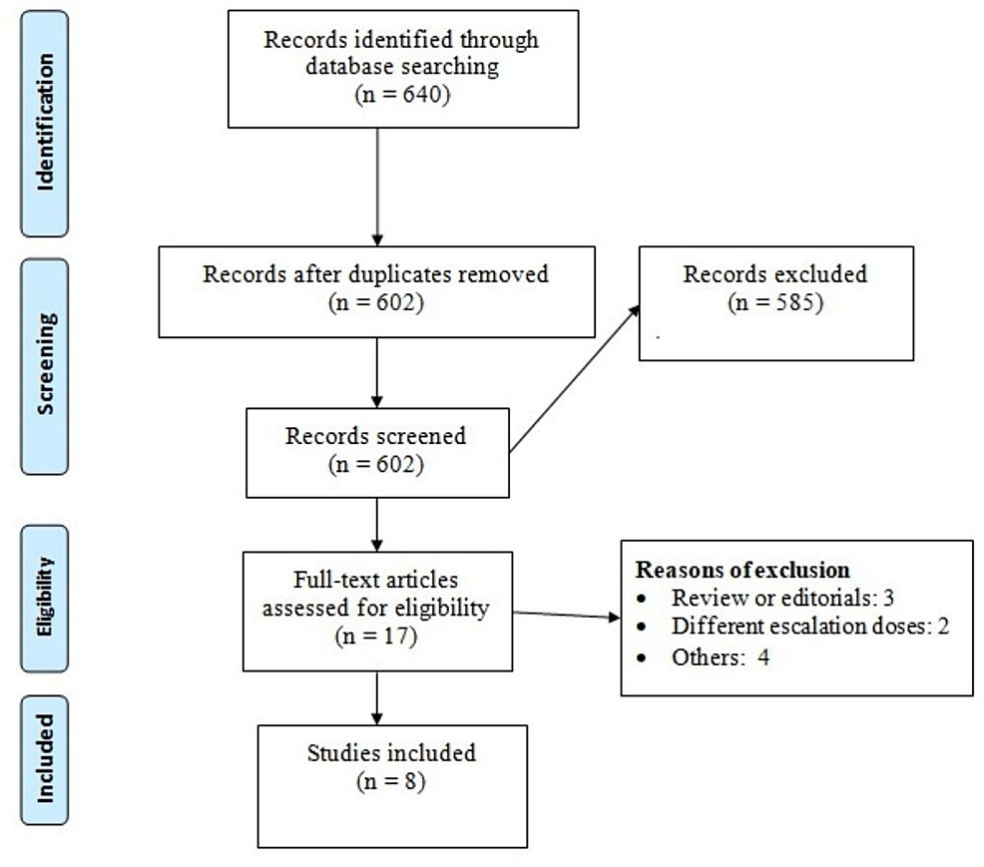
PRISMA flowchart PRISMA: Preferred Reporting Items for Systematic Reviews and Meta-Analyses

**Figure 2:**
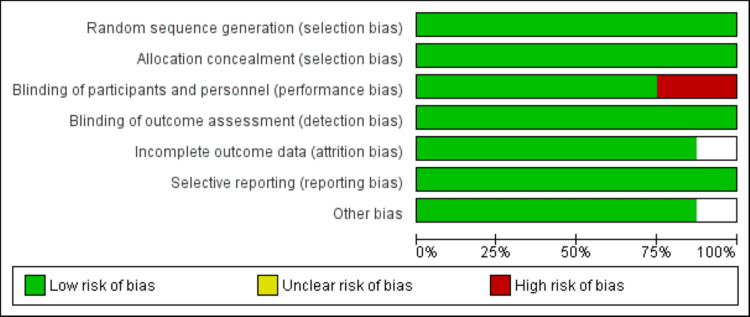
Quality assessment

Figure 1 shows the PRISMA flow diagram of the meta-analysis. Based on the online database search, 640 potentially eligible studies were identified. Of these 602 records were screened by abstract or title after removal of duplicates. After a detailed screening of the 17 full-text studies, eight met the inclusion criteria and were included in this meta-analysis [13-20]. Table 1 shows the characteristics of the included studies. Two studies assessed TZP as monotherapy, while six studies evaluated different TZP doses as add-on therapy. The follow-up of included studies ranged from 26 weeks to 72 weeks. Figure 2 shows the quality assessment of the included studies.

**Table 1:** Characteristics of included studies NR: Not reported

Author	Year	Therapy	Doses	Sample Size	Follow-up Duration	Age (Years)	Males (n)	Diabetes Duration (Years)
Dahl et al [13]	2022	Add-on therapy	5 mg	116	40 Weeks	62	61	14.1
			10 mg	119		60	72	12.6
			15 mg	120		61	65	13.7
Frias et al [14]	2018	Add-on therapy	5 mg	55	26 Weeks	57.9	34	8.9
			10 mg	51		56.5	30	7.9
			15 mg	53		56	22	8.5
Frias et al [15]	2021	Add-on therapy	5 mg	470	40 Weeks	56.3	220	9.1
			10 mg	469		57.2	238	8.4
			15 mg	470		55.9	214	8.7
Gao et al [16]	2023	Add-on therapy	5 mg	230	40 Weeks	53.1	134	7.4
			10 mg	228		53.5	126	7.9
			15 mg	229		54.3	129	7.6
Inagaki et al [17]	2022	Monotherapy	5 mg	159	52 Weeks	NR	NR	NR
			10 mg	158				
			15 mg	160				
Ludvik et al [18]	2021	Add-on therapy	5 mg	358	52 Weeks	57.2	200	8.5
			10 mg	360		57.4	195	8.4
			15 mg	359		57.5	194	8.5
Prato et al [19]	2021	Add-on therapy	5 mg	329	52 Weeks	62.9	198	9.8
			10 mg	328		63.7	209	10.6
			15 mg	338		63.7	203	10.4
Rosenstock et al [20]	2021	Monotherapy	5 mg	121	40 Weeks	54.1	56	4.6
			10 mg	121		55.8	72	4.9
			15 mg	121		52.9	63	4.8

Change in HbA1c (%) from Baseline

**Figure 3:**
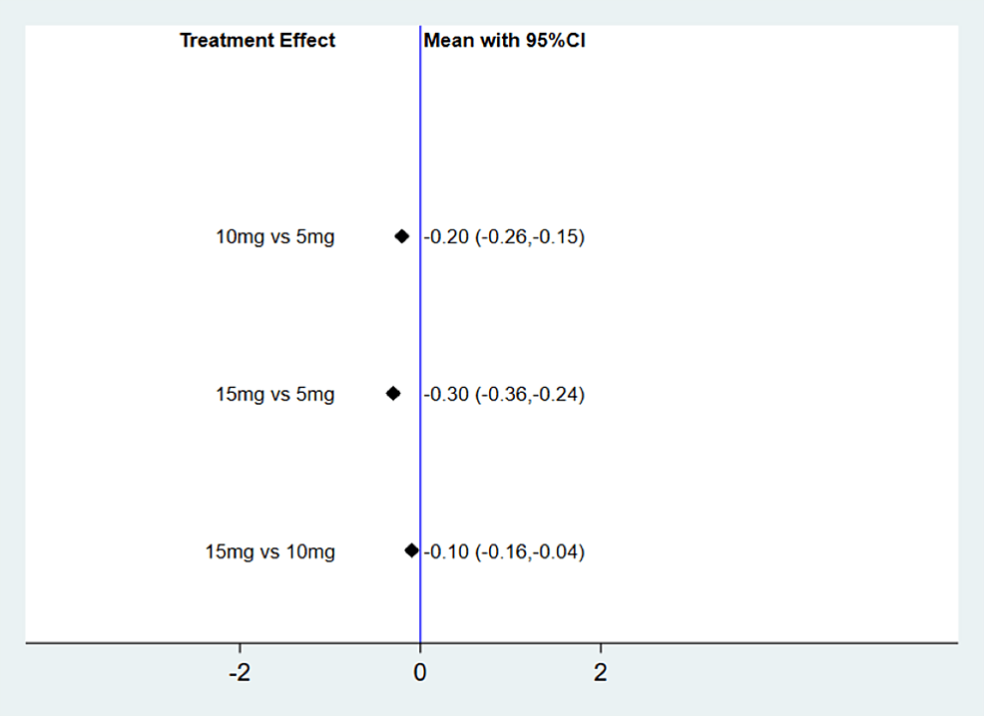
Change in HbA1c from baseline (%) CI: Confidence interval; HbA1C: Hemoglobin A1C

Figure 3 illustrates pairwise comparisons among different TZP doses. Compared to TZP 5 mg, the meta-analysis reveals that TZP 10 mg could significantly decrease HbA1c by 0.20% (mean difference {MD}: -0.20, 95% CI: -0.26, -0.15). Similarly, in comparison with TZP 5 mg, the meta-analysis demonstrates that TZP 15 mg could significantly reduce HbA1c by 0.30% (MD: -0.20, 95% CI: -0.36, -0.24). When compared with TZP 10 mg, the meta-analysis indicates that TZP 15 mg was able to significantly lower HbA1c by 0.10% (MD: -0.10, 95% CI: -0.16, -0.04). The SUCRA score highlights that in terms of reducing HbA1c, TZP 15 mg performed the best, followed by 10 mg and 5 mg, as depicted in Table 2.

**Table 2:** SUCRA score of each outcome SUCRA: Surface under the cumulative ranking curves; HbA1C:  glycated haemoglobin; FSG: Fasting serum glucose; SAE: Serious adverse events; AE: Adverse events; MACE: Major adverse cardiovascular events

Outcomes	5 mg	10 mg	15 mg
Hb1AC	0	0.5	1
Weight	0	0.5	1
FSG	0	0.5	1
SAE	0.2	0.5	0.8
MACE	0.3	0.4	0.9
AE	1	0.4	0.2

Change in Weight (Kg) from Baseline

**Figure 4:**
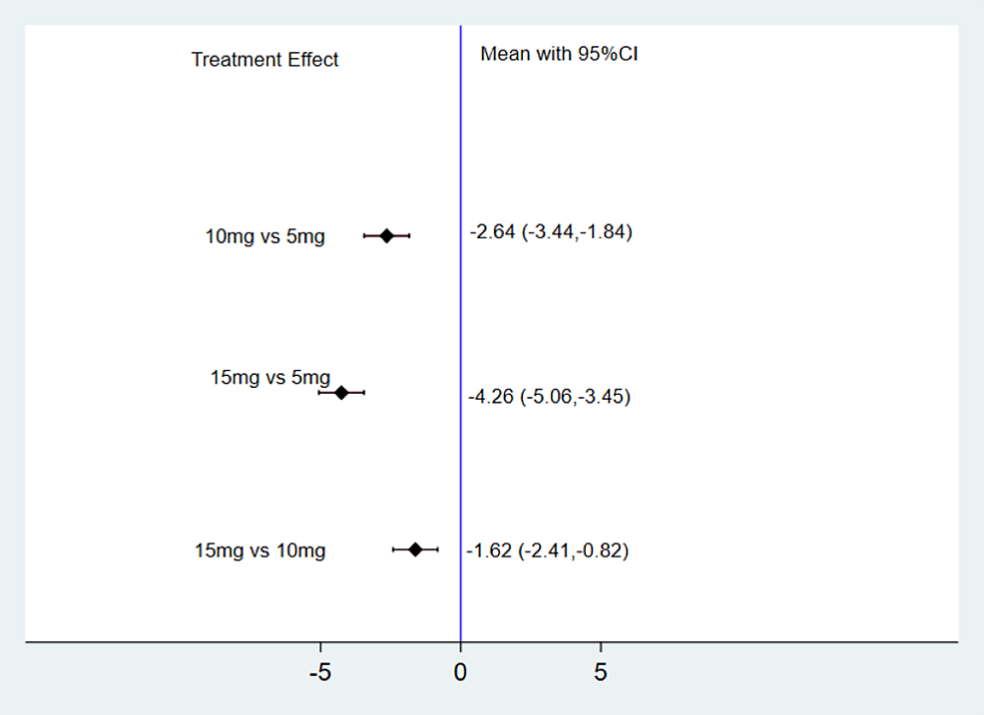
Change in weight from baseline (Kg) CI: Confidence interval

Figure 4 depicts the change in weight from baseline across different TZP doses. Compared to TZP 5 mg, the meta-analysis indicates that TZP 10 mg could significantly reduce weight by 2.64 Kg (MD: -2.64, 95% CI: -3.44, -1.84). Similarly, compared to TZP 5 mg, the meta-analysis demonstrates that TZP 15 mg could significantly decrease weight by 4.26 Kg (MD: -4.26, 95% CI: -5.06, -3.45). Moreover, in comparison with TZP 10 mg, the meta-analysis highlights that TZP 15 mg was effective in significantly lowering weight by 1.62 Kg (MD: -1.62, 95% CI: -2.41, -0.82). According to the SUCRA score, in terms of weight reduction, TZP 15 mg exhibited the highest effectiveness, followed by 10 mg and 5 mg, as indicated in Table 2.

Change in Fasting Serum Glucose (mg/dl) from Baseline

**Figure 5:**
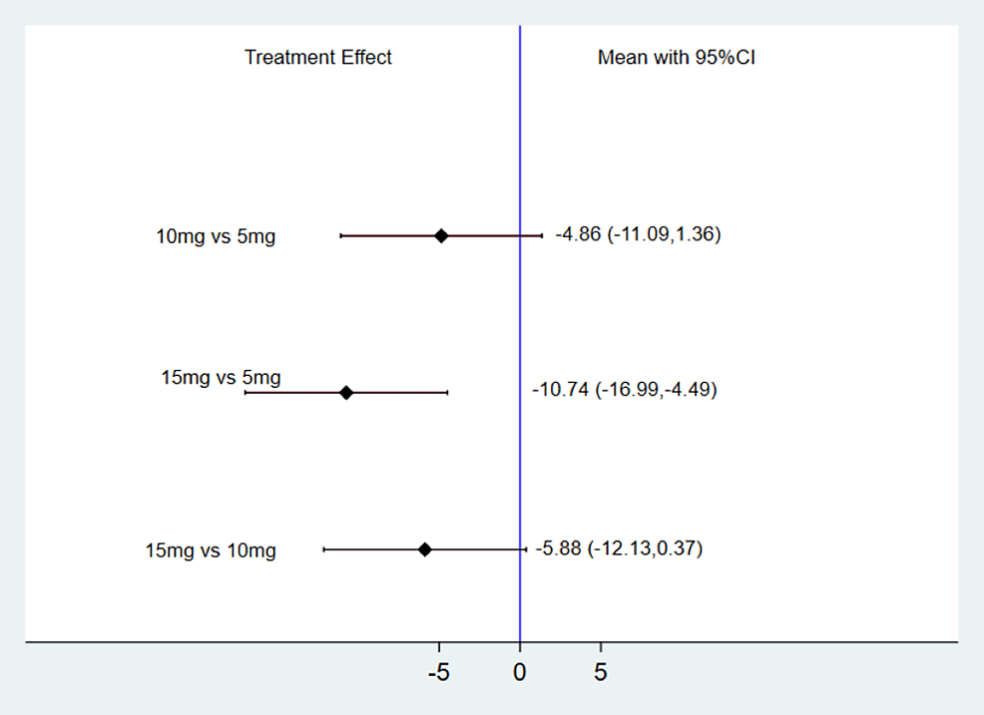
Change in FSG from baseline (mg/dl) CI: Confidence interval; FSG: Fasting serum glucose

Illustrated in Figure 5 is the alteration in fasting serum glucose (FSG) from baseline across various TZP doses. In comparison to TZP 5 mg, the meta-analysis demonstrates that TZP 10 mg exhibited a decrease in FSG by -4.86 mg/dl; however, this difference lacked statistical significance (MD: -4.86, 95% CI: -11.09, 1.36). Conversely, when compared to TZP 5 mg, the meta-analysis unequivocally highlights that TZP 15 mg led to a substantial reduction in FSG by 10.74 mg/dl (MD: -10.74, 95% CI: -16.99, -4.49). Furthermore, relative to TZP 10 mg, the meta-analysis provides evidence that TZP 15 mg was capable of significantly diminishing FSG by 5.88 mg/dl (MD: -5.88, 95% CI: -12.13, 0.37). The SUCRA score analysis underscores that TZP 15 mg excelled in terms of FSG reduction, followed by 10 mg and 5 mg, as depicted in Table 2.

Safety Analysis

Table 3 shows the risk of adverse events among three groups. Among all three doses, no significant differences existed among three doses regarding serious adverse events and MACE-4 events. However, the risk of adverse events was significantly higher in TZP 10 mg and 15 mg doses compared to TZP 5 mg. On the other hand, the risk of adverse events was not significantly different between TZP 10 mg and TZP 15 mg groups.

**Table 3:** Safety analysis SAE: Serious adverse events, AE: Adverse events, MACE: Major adverse cardiovascular events All values are presented as risk ratio (95% confidence interval)

Groups	SAE	MACE-4	AE
10 mg vs 5 mg	0.93 (0.73-1.20)	0.99 (0.57-1.74)	1.20 (1.10-1.43)
15 mg vs 5 mg	0.84 (0.65-1.09)	0.67 (0.36-1.25)	1.26 (1.05-1.50)
15 mg vs 10 mg	0.90 (0.69-1.17)	0.68 (0.36-1.28)	1.04 (0.87-1.25)

Discussion

The meta-analysis of eight RCTs with a pooled sample size of 5522 patients with diabetes found that among all the doses of TZP, the reduction in HbA1c, weight, and fasting serum glucose was significantly greater with a 15 mg dose compared to doses of 10 mg and 5 mg. With regard to serious adverse events, no significant difference was found among the three doses. This is the first meta-analysis that made direct comparisons between different doses of TZP. Previously, a meta-analysis conducted by Yu et al. [21] made pairwise comparisons. However, since this meta-analysis was performed, new studies have been conducted. The findings of our meta-analysis were consistent with the study conducted by Yu et al. [21] which reported a greater reduction in HbA1c and weight in the TZP 15 mg group, followed by the 10 mg and 5 mg groups.

The findings of this study show that within the 5 to 15 mg dose range, the impact of TZP in reducing weight and lowering glucose is dose-dependent, and TZP at 15 mg can achieve the greatest efficacy benefit in individuals with type 2 diabetes. It is vital to note that due to the presence of basal medications in the majority of the included studies, the beneficial results we obtained may be based on the context of the combination and not solely on the independent impact of TZP. Research indicates that TZP's ability to lower blood sugar levels is linked to heightened insulin sensitivity in individuals. Furthermore, the hypoglycemic impact of TZP becomes more pronounced as the dosage of TZP is increased. Both GLP-1 and gastric inhibitory polypeptide (GIP) positively influence insulin secretion [21]. TZP might decrease the metabolic requirement for insulin release by pancreatic β-cells by diminishing insulin resistance in individuals with type 2 diabetes. As a result, this could lead to a reduction in prolonged stress on β-cells [22]. In certain cases, the lowering of metabolic demands holds the possibility of reversing the impairment of islet β-cells for certain patients [22].

Different meta-analyses have demonstrated that TZP is more effective compared to insulin and GLP-1RA in decreasing weight and lowering glycemia, and does not increase the cardiovascular risk or the risk of adverse events, promising a new approach for the management of type 2 diabetes [23-24]. Clinical research has demonstrated that the impact of tirzepatide on regulating blood sugar levels is supported by simultaneous enhancements in the functioning of β‐cells, insulin sensitivity, and α‐cells. The administration of tirzepatide at a dose of 15 mg led to noteworthy enhancements in the secretion of insulin during both the initial and subsequent phases, as well as overall insulin secretion and sensitivity [22,25]. Additionally, during meal tolerance testing, tirzepatide exhibited the ability to decrease glucagon secretion both during fasting and in response to meals [26].

When comparing individuals achieving HbA1c < 7 in each dose of TZP, we found no significant differences among the three groups. For individuals with diabetes, an HbA1c level lower than 7% is important [27]. Striving to maintain HbA1c levels below 7% is a crucial aspect of diabetes care. It promotes better blood sugar control, reduces the risk of complications, and contributes to the long-term health and quality of life of individuals living with diabetes. Regarding the safety of different doses of TZP, no significant differences were found among the three groups in terms of the risk of serious adverse events and MACE events. However, the risk of adverse events was significantly higher in patients receiving TZP at 10 mg and 15 mg compared to 5 mg. However, the risk of adverse events was not significantly different between TZP at 10 mg and TZP at 15 mg.

Study limitations

The current meta-analysis has certain limitations. We found that TZP at 15 mg is more effective in reducing weight and HbA1c compared to the other two doses. However, we were unable to perform subgroup analysis due to a lack of individual-level data. It is important for future studies to thoroughly investigate the effects of various doses of TZP within diverse patient groups, encompassing individuals with hyperlipidemia, those with a prior history of cardiovascular events, and other relevant populations. Such research endeavors will significantly contribute to a more comprehensive understanding of how different dosages of TZP interact with these specific patient characteristics. By discerning the varying impacts of TZP doses within distinct subgroups, healthcare professionals will be better equipped to formulate precise and tailored dosage recommendations based on each patient's unique medical background and requirements. This personalized approach to dosage guidance will ultimately enhance the effectiveness and safety of TZP treatment strategies for a broad spectrum of patients. Another limitation of this research is the inadequate availability of concise short-term data. While assessing the enduring effectiveness of diverse TZP treatment doses depends on long-term evaluation, the value of assessing short-term effectiveness cannot be understated given the psychological impetus among individuals with T2DM to swiftly alleviate their glycemic levels. The studies encompassed in this analysis featured follow-up periods spanning 26 weeks or more, primarily yielding data on prolonged outcomes, and regrettably lacked immediate efficacy outcomes for TZP in the short term.

## Conclusions

In conclusion, this meta-analysis examined the efficacy and safety of different doses of tirzepatide (TZP) in individuals with type 2 diabetes. Our findings suggest that among the evaluated doses, TZP at 15 mg demonstrated superior effectiveness in reducing HbA1c, weight, and fasting serum glucose compared to doses of 10 mg and 5 mg. Notably, the reduction in HbA1c and weight showed a dose-dependent trend, with the 15 mg dose achieving the most substantial benefits. The safety analysis indicated that while serious adverse events and major adverse cardiovascular events (MACE) did not significantly differ among the three doses, the risk of overall adverse events was notably higher in the 10 mg and 15 mg TZP groups compared to the 5 mg group. In essence, this meta-analysis confirms TZP's potential as a promising therapeutic agent for type 2 diabetes management. Further research, incorporating diverse patient populations and longer-term assessments, will deepen our understanding of TZP's effects and refine its clinical applications, ultimately enhancing personalized treatment approaches and improving the quality of care for individuals with type 2 diabetes.
